# Smoke-free hospitals in Greece: Personnel perceptions, compliance and smoking habit

**DOI:** 10.1186/1617-9625-5-8

**Published:** 2009-03-31

**Authors:** Constantine I Vardavas, Izolde Bouloukaki, Manolis K Linardakis, Penelope Tzilepi, Nikos Tzanakis, Anthony G Kafatos

**Affiliations:** 1 Department of Social Medicine, Faculty of Medicine, University of Crete, Greece; 2 Department of Thoracic Medicine, University Hospital of Heraklion, Crete, Greece; 3 Department of Management, University Hospital of Heraklion, Crete, Greece

## Abstract

Smoke-free environments in Greece are scarce. Despite existent legislation that forbids smoking in all health care service centers, smoking is still evident. Using a random sample of hospital personnel from a large university hospital in Greece, we evaluated their smoking habits, perceptions and compliance towards hospital smoking regulations. 57.8% of the nursing personnel and 34.5% of medical/research staff were found to be current smokers (*p *< 0.05). Although 66% of the staff does not oppose the complete hospital smoking ban, 95% responded that they would prefer it to be partial. The above findings warrant the necessity for nurturing efforts to reduce smoking and increase the health professionals' awareness of their position as a role model to both patients and the society.

## Main text

Tobacco consumption is a leading cause of death and disability and a clear threat to global public health. In 2005, tobacco products were estimated to cause a death every six seconds and this number has been envisaged to increase if current trends prevail [1]. Comprehensive tobacco-control programmes that include smoke-free public places, smoking cessation services, price increases and mass media campaigns are just a number of tobacco control actions that can make the difference.

Since August 2002, in line with EU regulations, Greece has enacted legislation that prohibits smoking in all health care service centres such as public and private hospitals, health centres and pharmacies (Health Law 76017), but just as with the majority of relative legislations in Greece it is bluntly ignored by many [2]. Health care professionals can influence both tobacco consumption and provide vital information on its negative health effects; however their smoking habits and their beliefs on tobacco control measures are an indicator of the role they may play in the de-normalisation of tobacco use.

Taking the above into account, the purpose of our study is to investigate into hospital personnel (in a typical large regional hospital in Greece) with regards to their perceptions and compliance towards hospital smoking regulations and their current smoking habits.

## Methods

The sample population was selected from the University Hospital of Crete, which is located in Heraklion, Greece and provides primary and secondary care to the population of Heraklion and tertiary care to the population of Crete and the nearby islands. The hospital permanently employs 561 medical doctors and 480 nurses (1041 in total) out of which 10% were randomly selected (using the 2006 hospital personnel database and weighed according to the doctor/nurse ratio) and interviewed. Previous research in Greece has noted that the smoking prevalence among hospital staff is estimated at approximately 50% and although a higher number of respondents would be preferred, we aimed at repeatedly contacting all selected participants so as to eliminate the percentage that might not respond, as previous hospital based studies in Greece have not demonstrated very high participation rates [3,4]. Of the 104 participants randomly selected 100 were interviewed (96% response rate), with the male:female ratio of 1:2 and a mean age of 39.2 ± 7.4 years. The questionnaires were filled in by an experienced interviewer and collected data on their personal smoking habits and their opinions and perceptions with regard to smoking and exposure to second hand smoke (SHS) within the hospital premises. Descriptive measurements were used to define the characteristics of the personnel who participated in the study. Due to the small number of ex-smokers in the study group, non-smokers and ex-smokers were grouped together during the analysis and categorised as non-smokers. All *p*-values are based on two-sided tests and a significance level of <5% was designated. Continuous variables are presented as mean ± standard deviation, while qualitative variables were depicted with the use of frequencies. Student's t-test and a chi-square test (χ^2^) were used to calculate the distribution of the study group with regard to parameters such as occupation, gender, attitudes and level of smoking. The statistical analysis was performed using the statistical package SPSS 15.0.

## Results

As depicted in Table 1, a larger percentage of the nursing personnel were current smokers in comparison to the percentage of medical/research staff (57.8% vs. 34.5%, *p *< 0.05) while the nursing staff was also found to smoke more cigarettes per day (12 ± 11 vs. 6 ± 9, *p *< 0.05). No gender differences were noted, other than the number of years that the personnel smoked (male: 17.4 vs. female 13.5, *p *< 0.05).

**Table 1:** Smoking habits among a representative sample of hospital personnel in a large typical Greek hospital.

	**Medical-research staff**	**Nursing staff**	**Total**
	
	% (n)	% (n)	% (n)
Males	34.5 (19) ^‡^	31.1 (14)	**33.0 (33)**
Females	65.5 (36)	68.9 (31)	**67.0 (67)**
**Age **(years)	39.2 ± 7.1 ^†^	39.1 ± 7.8	**39.2 ± 7.4**
**Smoking habit**			
Smokers *	34.5 (19)	57.8 (26)	**45.0 (45)**
Ex-Non smokers**	65.5 (36)	42.2 (19)	**55.0 (55)**
**Years of smoking**	7.3 ± 9.1	9.4 ± 8.0	**8.0 ± 9.0**
***Cigarettes per day***			
*1–9 cigs/day*	15.8 (3)	3.8 (1)	**8.9 (4)**
*10–20 cigs/day*	63.2 (12)	73.1 (19)	**68.9 (31)**
*>20 cigs/day*	21.1 (4)	23.1 (6)	**22.2 (10)**
Mean cigarettes per day *	6 ± 9	12 ± 11	**8 ± 11**

‡ Values are N (%). Chi-square test (χ^2^).

† Values are mean ± standard deviation. Student t test.

*p-value < 0.05

** Ex-Non smokers were defined as those who currently are not smokers regardless of if they smoked in the past or not.

Table 2 mainly presents the hospital personnel's attitudes towards the smoking ban and their self-reported exposure to second hand smoke (SHS). Although 70.9% of the medical staff and 60% of the nursing staff approve of the complete hospital smoking ban, all current smokers (100%) reported smoking daily at work, thus flagrantly ignoring the specific legislation that forbids smoking within the hospital premises even though almost half of the smokers support the idea of the complete smoking ban. Additionally, fewer female non-smokers approved of smoking at work in comparison to males (42.4% vs. 58.3%, respectively). Irrespective of gender, those who found SHS exposure annoying in the hospital were also more likely to disapprove of smoking in the hospital (*p *< 0.001) and when asked if the complete smoking ban should change into a partial (with designated smoking and non-smoking areas inside the hospital) 93.3% of smokers and 96.4% of non-smokers responded that they would prefer such legislation.

**Table 2:** Hospital personnel's attitude towards a smoke-free hospital policy in Greece.

**Variable**		**Medical-research staff**	**Nursing staff**	**Total**
		
		% (n)	% (n)	% (n)
**Smoke at work**				

% of total personnel		34.5 (19)	57.8 (26)	**45 (45)**
% of smokers		100 (19)	100 (26)	**100 (45)**

**Your attitude towards smoke-free hospitals?**				

Total personnel	*Approve*	70.9 (39)	60.0 (27)	**66 (66)**
	*Disapprove*	29.1 (16)	40.0 (18)	**34 (34)**
Smokers	*Approve*	52.6 (10)	42.3 (11)	**46.7 (21)**
	*Disapprove*	47.4 (9)	57.7 (15)	**53.3 (24)**
Non smokers **	*Approve*	80.6 (29)	84.2 (16)	**81.8 (45)**
	*Disapprove*	19.4 (7)	15.8 (3)	**18.2 (10)**

**How often are you exposed to SHS at work?**				

Non smokers **	Daily-Almost daily	66.7 (24)	73.7 (14)	**69.1 (38)**
	*Half of the days*	22.2 (8)	10.5 (2)	**18.2 (10)**
	*Almost never*	11.1 (4)	15.8 (3)	**12.7 (7)**

**Does cigarette smoke during work annoy you?**				

Non smokers **	*Yes*	80.6 (29)	84.2 (16)	**81.8 (45)**
	*No*	19.4 (7)	15.8 (3)	**18.2 (10)**

*p-value < 0.05

** Non smokers were defined as those who currently are not smokers regardless of if they smoked in the past or not.

## Discussion

Our results in regards to smoking habits are very similar to those found in previous studies amongst hospital staff in Greece, which are characterised mainly by the elevated smoking rates of both physicians and nursing staff, with the highest prevalence found among the latter. These findings indicate the unwillingness of the Greek health care professional to fulfil their role as an example towards both patients and the society, and implicate their inability to effectively advocate for tobacco control measures and smoking cessation for the reduction and prevention of the plethora of diseases tobacco is responsible for [3,4].

Achieving smoke free hospitals is notoriously difficult. Research among UK hospitals also revealed that smoking is often noticed despite relevant regulation and policy enforcement is often inadequate [5] while other studies have also revealed the personnel's similar preference to partial smoking bans and the reluctance of personnel to challenge personnel, visitors and patients to stop smoking on the hospital site [6-8]. Although designated smoking areas of a partial ban might reduce non-smokers exposure to SHS, since smokers might be motivated/encouraged to use the smoking rooms, there is an imminent danger of regression towards ignoring even this partial measure and dramatically increase the already elevated levels of measured SHS exposure within Greek hospitals [9].

As complete smoking bans, can and have been shown to influence and increase personnel's quitting attempts, [7,10] encourage patient smoking cessation [11] and reduce exposure to SHS it is imperative that health practitioners, policy makers and hospital management boards nurture and enhance efforts that aim to reduce smoking among health professionals and support smoke free environments. As smoking plays an important part in health care expenditure and health care service utilization it is in the interest of health care professionals, to hold a key position in the development of the overall public health policy [12].

Taking into account the addictive properties of nicotine and the elevated smoking rates of both nurses and physicians in Greece, health professional oriented educational programmes in treating nicotine addiction, the creation and support of hospital-based smoking cessation centres and the continuous monitoring of compliance to existing legislations should be central to efforts that reinforce the viability of a smoke-free health care system. Further research into the factors that modify both personnel smoking habits and the health professionals' beliefs on tobacco related issues is warranted.

## Competing interests

The authors declare that they have no competing interests.

## Authors' contributions

CIV, conceived the idea and had the main role in manuscript preparation while authors IB and PT participated in data collection and study design, author MKL performed the statistical analysis and authors NT and AGK participated in study design, coordination and data interpretation. All authors participated in manuscript preparation and agree on its content.
